# P01-047 – PH with right-sided heart failure in FMF

**DOI:** 10.1186/1546-0096-11-S1-A50

**Published:** 2013-11-08

**Authors:** AV Sargsyan, MZ Narimanyan

**Affiliations:** 1 Internal Medicine, Yerevan, Armenia; 2 Family Medicine, Yerevan State Medical University, Yerevan, Armenia

## Introduction

FMF is associated with pulmonary hypertension(PH) due to amyloidosis. However, clinically overt PH with right-sided heart failure remains a rare event limited to few patients with pulmonary amyloidosis secondary to FMF. We report two cases of FMF patients, with and without amyloidosis, who experienced PH with right-sided heart failure. To our knowledge, this is the first case report on cor pulmonale in FMF patient without amyloidosis.

## Case report

### First

A.R.,72 year old non-smoker male presented with 6-month history of cough, dyspnea and intensive weight loss (8 kg). He had typical FMF abdominal and chest attacks since the age of 28. He took colchicine irregularly,during attacks only. He gave a positive family history. His daughter (40,has had FMF attacks since the age of 35) and cousin brothers were also affected. No history of occupational exposure. Series of X-ray and thoracic CT scan demonstrated unilateral ground-glass opacities in a basal segment of right lung and cardiomegaly. Echocardiography showed hypertrophy of left ventricle, dilated right ventricle, tricuspid regurgitation, and PH, findings compatible with cor pulmonale. Upper GI endoscopy with gastric biopsy revealed atrophic gastritis. Laboratory investigations revealed: CRP 79.2mg/L, SAA 210g/L, creatinin 162.1-189μmol/l and proteinuria 0.19g/daily. Two months later patient died of heart failure. Postmortem examination showed emphysema, lung fibrosis and sclerosis, myocardial hypertrophy and kidney arteriolosclerosis. Daughter’s genetic test revealed one mutation in exon 2 in the heterozygous state E148Q/N.

### Second

S.H.,47 year old male, a smoker, presented with cough, progressive dyspnea and pedal edema. He had typical FMF abdominal and chest attacks since early childhood. He gave a positive family history. His sister and brother were also affected. He took colchicine irregularly, during attacks only. Genetic test showed compound heterozygosity (M694V M680IG/C). ElectroKG showed atrial fibrillation and low QRS voltage in the limb leads. Doppler ECG demonstrated biventricular wall thickening, dilated right ventricle, diastolic dysfunction, and pulmonary hypertension. Laboratory investigations revealed: creatinin 172.4μmol/l, proteinuria 1.4g/daily. CT demonstrated diffuse interstitial lung infiltrates and bullas (marked air cysts). Eight months later, patient died of heart failure. Postmortem examination showed extensive deposits of amyloid in the kidneys, pulmonary vasculature, alveolar septa, pleura and myocardium.

## Discussion

FMF patients with chest attacks may develop PH. If present, PH is a sign of advanced disease, and the survival rate after diagnosis is low. A diagnosis of PH should be considered in patients with and without amyloidosis and unexplained dyspnea or fluid overload. Although pulmonary involvement may occur in FMF, PH with right sided heart failure is considered an infrequent diagnosis and is rarely the cause of death.

## Disclosure of interest

A. Sargsyan Consultant for: clinical and lab tests, M. Narimanyan Consultant for: clinical and lab tests.
